# Hypertensive Urgencies Following Percutaneous Adrenal Mass Biopsy in a Day Surgery Unit: A Case Report

**DOI:** 10.7759/cureus.80568

**Published:** 2025-03-14

**Authors:** Dongmei Peng, Zhichao Li, Li Hou, Sha Ouyang

**Affiliations:** 1 Day Surgery Center, West China Hospital of Sichuan University, Chengdu, CHN; 2 Day Surgery Center, Chengdu Shang Jin Nan Fu Hospital / Shang Jin Hospital of West China Hospital, Sichuan University, Chengdu, CHN

**Keywords:** adrenal mass biopsy, case report, day surgery, hypertensive crisis, hypertensive urgencies, postoperative management

## Abstract

We present a case of a 59-year-old female who developed hypertensive urgencies following a percutaneous biopsy of an adrenal mass in a day surgery unit. The patient, with no prior history of hypertension, experienced a significant blood pressure elevation post-procedure, necessitating urgent antihypertensive management. This case underscores the importance of vigilant preoperative evaluation to exclude pheochromocytoma and highlights the potential risks associated with adrenal mass biopsies, particularly in the context of undiagnosed pheochromocytoma.

## Introduction

Pheochromocytoma, a rare neuroendocrine tumor originating from the adrenal medulla, is characterized by excessive catecholamine secretion, leading to episodic hypertension, headaches, and palpitations. However, atypical presentations, such as normotension, isolated symptoms, or incidental findings on imaging, can complicate diagnosis, as seen in this case. Mechanical manipulation of an unsuspected pheochromocytoma can trigger a massive release of catecholamines, resulting in a sudden and severe hypertensive crisis. Percutaneous biopsy of adrenal masses is a critical diagnostic tool, especially for distinguishing between benign and malignant lesions. Despite advancements in imaging and biopsy techniques, complications such as hypertensive crises remain a significant concern, particularly when pheochromocytoma has not been excluded preoperatively [1].

## Case presentation

**Figure 1:**
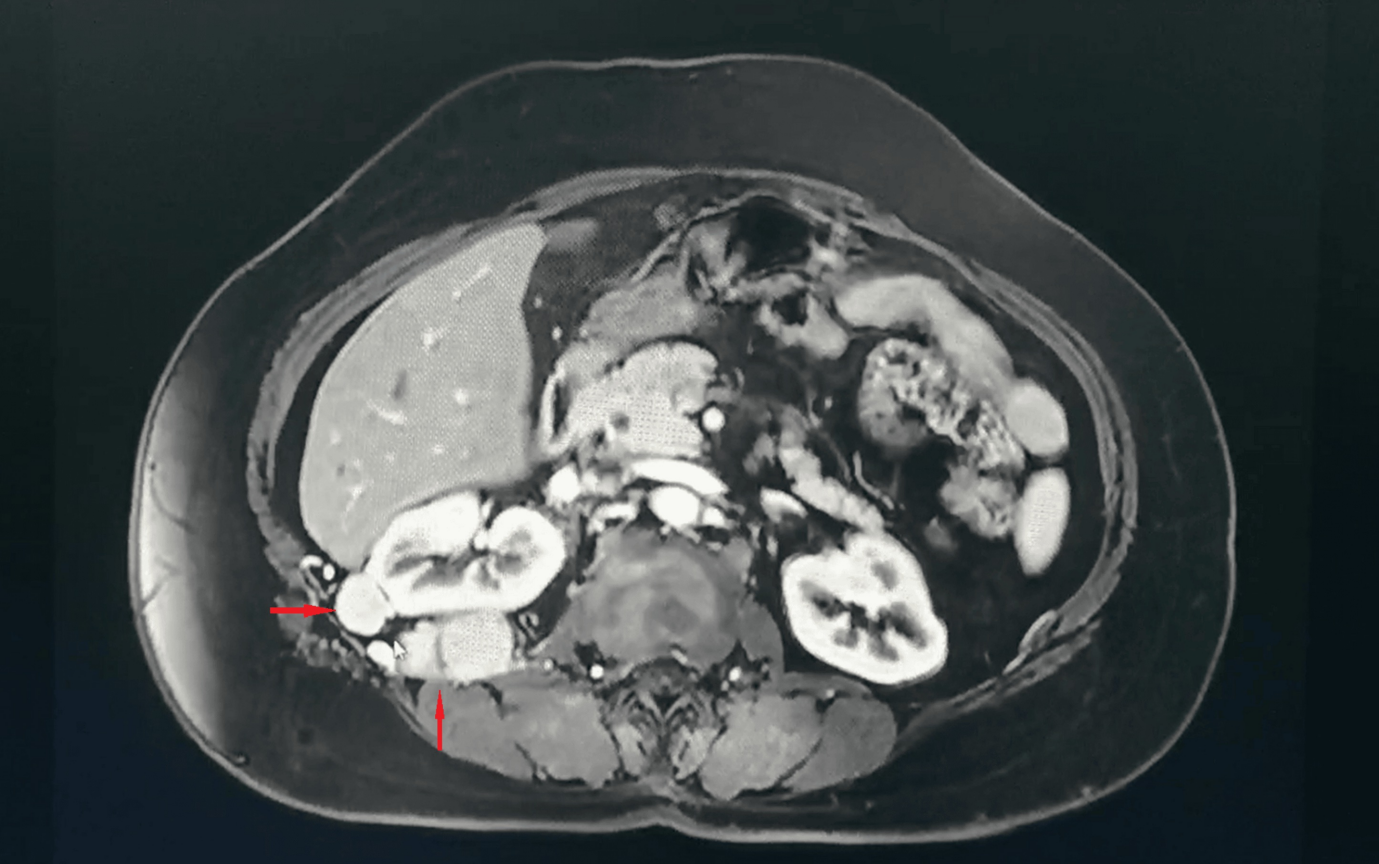
Abdominal MRI findings. The red arrows indicate multiple perinephric masses.

**Figure 2:**
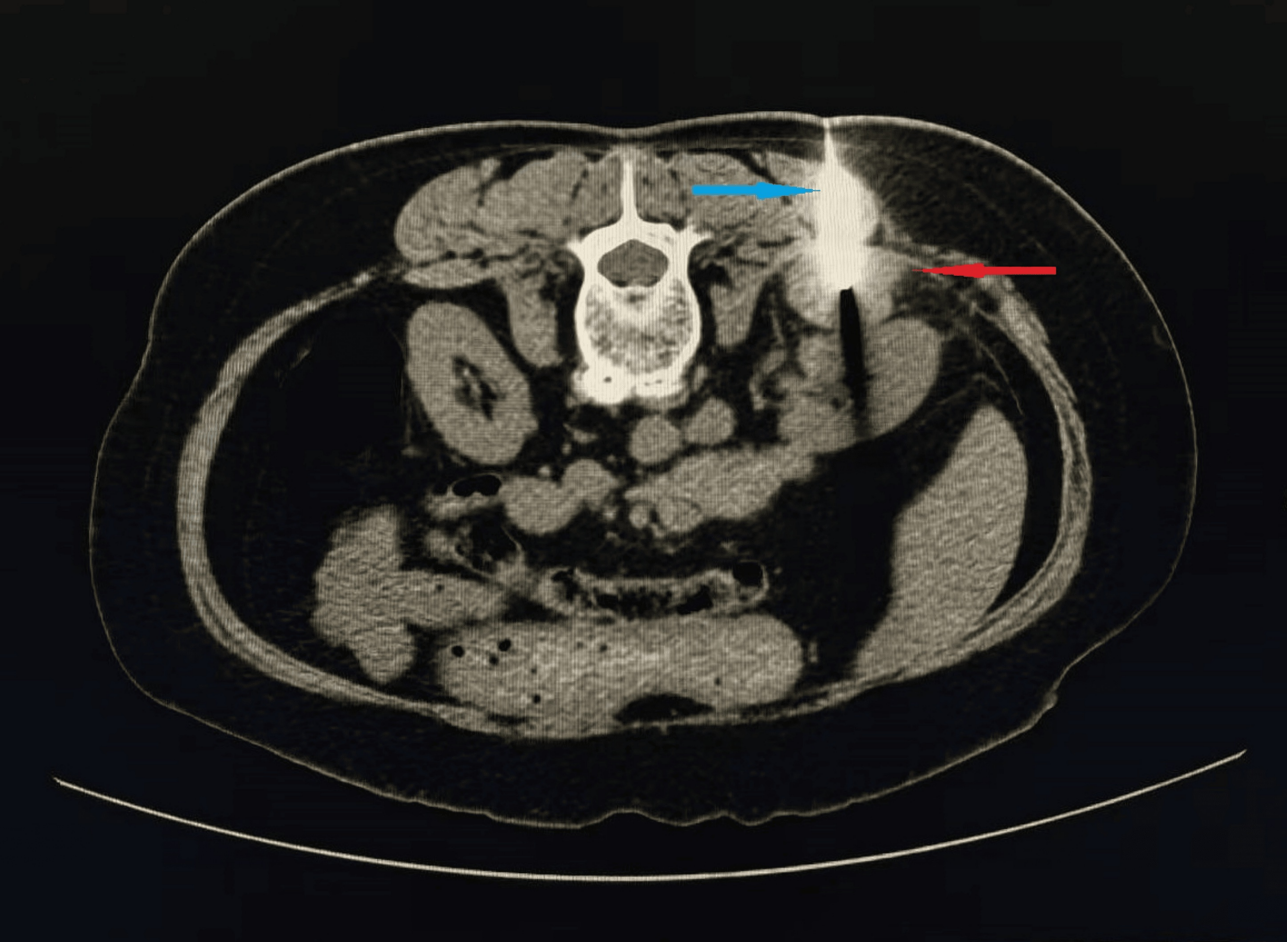
Image of a CT-guided percutaneous adrenal mass biopsy. The red arrow indicates the biopsied mass, and the blue arrow indicates the biopsy needle.

A 59-year-old female was admitted to our day surgery unit on January 9, 2025, for the evaluation of a right adrenal mass discovered incidentally one month prior to presentation. The patient had a history of surgical treatment for a right renal cyst and left renal stones. Initial blood pressure was 117/66 mmHg. Preoperative MRI (Figure 1) showed that the right kidney demonstrated normal size and morphology. Multiple nodules and masses with isointense T1 and hyperintense T2 signals were observed in the anterior portion of the right kidney, perirenal region, posterior to the inferior vena cava, and posterior right abdominal wall. The largest lesion was located posterior to the right kidney, measuring 3.7×2.5 cm, showing restricted diffusion and heterogeneous enhancement on contrast-enhanced imaging. The lesions exhibited ill-defined margins with the right diaphragm, posterior inferior segment of the right hepatic lobe, posterior right abdominal wall, right retroperitoneum, and right perirenal fascia. Compression of the inferior vena cava was noted, along with thickening of the right retroperitoneum, right perirenal fascia, and bridging septa. The right adrenal gland was poorly visualized. Under local anesthesia, a CT-guided percutaneous biopsy of the adrenal mass (Figure 2) was performed without immediate complications. Postoperatively, the patient’s blood pressure increased to 188/90 mmHg within two hours, prompting administration of sublingual nitroglycerin and oral prazosin. Despite initial stabilization, the patient returned the following day with severe hypertension (203/91 mmHg), dizziness, and nausea, diagnosed as a hypertensive emergency. Emergency treatment with nicardipine and nifedipine controlled the blood pressure, and the patient was discharged on oral amlodipine.

## Discussion

This case highlights the diagnostic challenges and potential complications associated with adrenal mass biopsies, emphasizing the critical importance of excluding pheochromocytoma preoperatively. The patient’s lack of typical symptoms and nonspecific imaging findings delayed the preoperative suspicion of pheochromocytoma. The hypertensive crisis post-biopsy underscores the need for careful preoperative evaluation, including mandatory biochemical screening for catecholamine excess, even in asymptomatic patients [2-3].

The use of α-adrenergic blockade prior to biopsy remains controversial. While some studies suggest that preoperative α-blockade may not significantly improve perioperative outcomes [4-6], others advocate for its use to mitigate the risk of catecholamine-induced hypertensive crises [7-8]. In this case, the rapid administration of α-blockers and calcium channel blockers effectively managed the hypertensive urgencies, preventing target organ damage. However, the absence of preoperative biochemical screening for pheochromocytoma represents a significant oversight, which could have prevented the hypertensive crisis.

The day surgery model, while offering significant advantages in terms of efficiency and cost-effectiveness [9-10], presents unique challenges in managing postoperative complications, particularly in procedures involving delicate endocrine organs. This case highlights the critical need for robust follow-up protocols and comprehensive emergency response plans for patients undergoing adrenal biopsies in day surgery units. Our findings suggest that the current standard of care may be insufficient in addressing the unique physiological challenges posed by adrenal procedures in an ambulatory setting. The implementation of enhanced preoperative protocols, including mandatory biochemical screening for pheochromocytoma, is essential to mitigate these risks. Furthermore, the development of specialized training programs for day surgery staff, focusing on the recognition and management of adrenal-related complications, is essential to ensure patient safety. This case underscores the importance of balancing the efficiency of day surgery models with the need for meticulous perioperative care, particularly in procedures involving endocrine organs. Future research should focus on developing evidence-based guidelines for adrenal biopsies in ambulatory settings, incorporating risk stratification models and standardized follow-up schedules to optimize patient outcomes.

## Conclusions

Percutaneous biopsy of adrenal masses is a valuable diagnostic tool but carries significant risks, particularly when pheochromocytoma has not been excluded preoperatively. Vigilant perioperative monitoring, mandatory preoperative biochemical screening for pheochromocytoma, prompt recognition of complications, and effective management strategies are essential to ensure patient safety. Further research is needed to optimize the care of patients undergoing adrenal biopsies in day surgery units, with a focus on preoperative risk assessment and exclusion of pheochromocytoma.
